# Oncolytic Adenoviruses in Gastrointestinal Cancers

**DOI:** 10.3390/biomedicines6010033

**Published:** 2018-03-11

**Authors:** Raquel T. Yokoda, Bolni M. Nagalo, Mitesh J. Borad

**Affiliations:** 1 Department of Medicine, Division of Hematology Oncology, Mayo Clinic Arizona, 13400 E Shea Blvd, Scottsdale, AZ 85205, USA; Yokoda.Raquel@mayo.edu (R.T.Y.); Nagalo.Bolni@mayo.edu (B.M.N.); 2 Department of Molecular Medicine, Mayo Clinic, Rochester, MN 55905, USA; 3 Center for Individualized Medicine, Mayo Clinic, 200 1st St SW, Rochester, MN 55905, USA; 4 Mayo Clinic Cancer Center, 5881 E Mayo Blvd, Phoenix, AZ 85054, USA

**Keywords:** adenovirus, oncovirotherapy, assessment of adeno-vector safety, gastrointestinal cancers, oncolytic therapy

## Abstract

Gastrointestinal malignancies are challenging cancers with considerable economic and societal impacts on health care systems worldwide. While advances in surgical approaches have provided benefits to a proportion of patients, only modest improvements have been attained in the treatment of patients with advanced disease, resulting in limited improvement in survival rates in these patients. Oncolytic adenoviruses are being developed to address gastrointestinal malignancies. Each platform has evolved to maximize tumor-cell killing potency while minimizing toxicities. Tumor-specific bioengineered adenoviruses using chimeric promoters, prodrug convertase enzymes, lethal genes, tumor suppressor genes, and pseudo-typed capsids can provide the innovations for eventual success of oncolytic virotherapy. This article will review the developments in adenoviral platforms in the context of specific gastrointestinal cancers. From the bench to the implementation of clinical trials, this review aims to highlight advances in the field from its early days to the current state of affairs as it pertains to the application of adenoviral oncolytic therapy to gastrointestinal cancers.

## 1. Introduction

Gastrointestinal cancers are a significant public health concern worldwide. They have a considerable impact on health economics that permeates multiple aspects of healthcare ranging from screening and prevention to hospice care [1]. In the United States, colorectal cancer is the fourth leading cause of cancer mortality. Pancreatic cancer and hepatocellular cancer also feature in the ten most lethal cancers in the United States [2]. Given the tremendous unmet therapeutic need for these cancers, novel approaches are in imminent need. Oncovirotherapy using adenoviruses (Ads) represents a very attractive anti-cancer therapeutic platform.

This review will focus on the evolution of adenoviral bioengineering and manipulation in esophageal, gastric, pancreatic, liver, biliary, and colorectal cancers. Progress in the field from pre-clinical studies to initial clinical trials will be covered with emphasis on barriers in the area and lessons learned to date. It is important to highlight that gastrointestinal malignancies were grouped as a way to facilitate the understanding of the evolution of the viral vectors per gastrointestinal organ. The cell biology of a vast heterogeneity of tumors in each organ is beyond the scope of this review.

## 2. Oncolytic Adenoviral Platform Development

Ads are non-enveloped DNA viruses with an icosahedral capsid encompassing a linear duplex genome of ~36 kb. Ads have been found in the majority of vertebrates [3]. Human Ads are ubiquitous in the environment and have been classified into 57 serotypes (Ad1-Ad57) based on cross-susceptibility to neutralizing antibodies and seven subgroups (A–G). Within each subgroup, there are similarities in virulence and tissue tropism [4]. In immunocompetent individuals, human Ad infections are mild, consisting of self-limited respiratory cold-like infections. 

Human adenoviruses represent an interesting oncolytic virotherapy platform given their (1) high transduction efficiency in transformed cells (~10,000 viral particles per infected cell), (2) lack of integration into the host genome resulting in a lowered risk of insertional mutagenesis, (3) low seroprevalence with regards to specific serotypes, (4) high fidelity DNA polymerase, which confers relative stability, and (5) ability to attain tumor specificity through substitution of the viral promoter with cancer tissue selective promoters or mutations that enable virus replication to occur preferentially in transformed cells.

Oncolytic virotherapy (OV) is a promising therapeutic platform with applications across a broad array of malignancies. The field of OV has its roots in 1890 when the first reports of spontaneous tumor regression were noted, following an episode of viral illness in a patient with leukemia. Similar observations followed in patients exposed to viral infections such as varicella, hepatitis, and measles in patients with Hodgkin’s disease, resulting in spontaneous regressions. However, these effects were mostly transient in nature. After a period of relative dormancy, a revival in the OV arena over the last 15 years has been incited by the increased understanding of the biology of viruses and advances in synthetic biology and recombinant nucleic acid technology allowing for the synthesis of more potent, selective and safe therapeutic viral vectors. In 2006, the adenovirus H101 was approved for clinical use in China for patients with advanced nasopharyngeal cancer [5]. Thus far, a series of clinical trials using H101 combined with standard chemotherapy demonstrated a better overall response in head and neck cancers [6].

Oncolytic adenoviral (OA) strategies have been evaluated in gastrointestinal (GI) malignancies. Although the initial research was conducted with Ad5, natural tropism and seroprevalence are becoming critical factors for vector success. A number of Ad subgroups can provide natural GI tropism such as the subgroup A (Ad12), and subgroup F (Ad40, Ad 41) [3]. The Ad12 E1B protein had similarities with A-gliadin and was studied as a factor in the development of gluten intolerance [7]. Ad40 and Ad41 are responsible for acute diarrhea and gastroenteritis, mostly in children [8]. These Ads use coxsackie-adenovirus receptors, CAR, and Ad 41 may promote disruption of the enterochromaffin cells and enteric glial cells leading to serotonin release [9]. Other types of natural tropism [3] are described in Table 1.

Additionally, tumor specificity and tropism can be enhanced through promoter driven replication. In GI malignancies, a number of adenoviral tumor-specific promoters have been analyzed to date (Table 2). Each of them will be discussed in the tumor-type-specific sections that follow.

Ads provide the possibility for hexon swapping and fiber pseudotyping among the subgroups, which is attractive toward maximizing targeting and delaying immune neutralization. Given this vast array of Ad serotypes, chimeric capsid investigations are still evolving, especially when considering which combinations would best enhance tissue tropism and provide on-target attachment. A few studies have shown that some chimeras combined with specific molecules could promote a reduction of tropism, which is of significant interest, as it may enhance virus bioavailability and contribute to diminishing liver and reticuloendothelial system sequestration [19].

## 3. Preclinical Perspectives in Gastrointestinal Cancers

### 3.1. Esophageal Cancer

Esophageal cancer has an incidence of 17,290 cases a year in the United States (USA), and patients who are inflicted typically experience a reduced quality of life [20]. Although there have been a number of advances in treatment in recent years, the overall mortality remains considerably high. Oncolytic Ads have undergone pre-clinical evaluation in esophageal cancer models (Table 3). One of the first such Ad vectors was designed to induce cell cycle arrest, which enhanced the oncolytic effect, with reduced nuclear factor kappa-B (NF-κB) and maximized apoptosis, primarily in p53 mutant cells [21].

Meanwhile, another study evaluated resistant tumor cells which exhibit properties of cancer stem cells. Radiotherapy resistance is a significant concern in esophageal cancer. The study analyzed an Ad carrying the tumor necrosis factor (TNF)-related apoptosis-inducing ligand (TRAIL) that preferentially induced apoptosis in radio-resistant cancer cells [22]. Another group investigated the combined effects of chemotherapy and oncovirotherapy using Ad. Four drugs were analyzed in combination with a modified Ad5, which encompassed the deletion of the 55 kDa-encoding *E1B* region as and a part of the E3 region. The four medications tested were 5-fluorouracil, etoposide, mitomycin C, and cisplatin. 5-Fluorouracil intensified the cell cycle at the S phase and promoted G2/M phase entry. Conversely, cisplatin produced G1 phase arrest. Interestingly, cisplatin action was antagonistic to the Ad vector, as it inhibited the Ad-mediated cell-cycle, not favoring a viral cytopathic effect. As such, cisplatin could lead to cross-resistance with Ad vectors [23].

Furthermore, another group developed the vector Telomelysin (OBP-301), which is an Ad5, with *E1A* genes under the control of a human telomerase reverse transcriptase (hTERT) promoter. The study achieved selective viral replication in tumor cells that expressed telomerase activity. The pre-clinical efficacy of this vector was found to be encouraging, and its evaluation has now progressed to clinical trials [24]. Along these lines, another group tested the adenoviral vector H101 in esophageal carcinoma [25]. 

A chimeric vector, using Ad H101 as a packaging template and the Newcastle disease virus (NDV) hemagglutinin-neuraminidase (HN), was tested in animal models. An increase in reactive oxygen species resulted in cytotoxicity and complete curative responses with prolonged survival with the use of intra-tumoral vector inoculation [26]. Despite being attractive tools conceptually, chimeric vectors have demonstrated diminished transduction rates when compared to wild types, resulting in a somewhat tempered enthusiasm for further evaluation.

### 3.2. Gastric Cancer

Gastric cancer has an incidence of 26,240 cases per year in the USA [20]. Surgery can be curative, but diagnosis in the setting of patients with advanced stage limits the number of patients eligible for surgical therapies. Novel therapeutic approaches are needed for patients with advanced disease.

OV therapy represents a promising platform for use in gastric cancer (Table 4). Enhancements in safety can be achieved by manipulation using Ad tumor-specific promoters, which enable the development of conditionally replicating adenoviruses (CRAds). Midkine (MK) and cyclooxygenase-2 (Cox-2M and Cox-2L) demonstrate high transcriptional activity in cell-lines from gastric cancer and represent promising elements for incorporation into CRAds. A study using an Ad5/3 vector with the Cox-2 promoter (Cox-2CRAd) demonstrated encouraging anti-tumor activity in gastric cancer models [11]. In another study, a CRAd using the CEA promoter was evaluated in gastric cancer cell lines with high CEA and had potent efficacy [17].

Along these lines, a group investigated the Ad Telomelysin (OBP-301), to target quiescent stem-like cells. These cells are in a dormant phase, which makes them resistant to chemotherapies. A strategy to overcome their resistance is to mobilize them into the cell cycle to elicit a treatment response. It has been shown that cell cycle mobilization can be induced by adenoviral infection. This study was important from this perspective, given that mobilization of cells from a quiescent state into the cell-cycle had only been previously achieved in leukemia [14].

Another study established a viral gene therapy approach by creating an Ad with endostatin, which is known to be an inhibitor of angiogenesis [27]. This vector exerted a compounded effect of the wild-type OV and endostatin toward promoting oncolysis. Correspondingly, another study investigated an Ad OV platform under a survivin promoter along with the Hsp-70 chaperone gene. The vector replicated selectively only in survivin-positive gastric cancer cells in mouse models [15].

### 3.3. Liver and Biliary Cancers

Liver and intrahepatic bile duct cancers have an incidence of approximately 42,220 cases per year in the USA [20]. Hepatocellular carcinoma (HCC) is the most common primary liver cancer [28]. Current therapies for advanced HCC have thus far not resulted in a high proportion of cures. The application of OV is an attractive approach in this setting, and a number of pre-clinical evaluations have been undertaken in this context (Table 5). 

Ad CV890 was the first Ad5-specific developed for selectively targeting hepatocellular carcinoma (HCC) by using alpha-fetoprotein (AFP) as a tumor-specific promoter. This study evaluated the ability of the vector to eliminate distant tumor recurrence when used in combination with doxorubicin in animal models. These promising data support further evaluation of this OV in clinical trials [29].

A pioneer Ad in OV, ONYX-015 [30], has been evaluated in HCC. Its safety profile and oncolytic efficacy were encouraging in animal models where the vector was loaded with a murine endostatin gene [31].

Another strategy for enhancing OV and gene therapy delivery is the dual promoter approach. It can enhance tumor specificity as well as vector delivery of gene therapy in HCC. A CRAd system placed the Ad *E1B* gene under a *TP53* gene expressing cassette coupled with hTERT promoter and a hypoxia response element (HRE) promoter. The goal was to foster p53 protein production in a hypoxic microenvironment in telomerase-positive HCC cells. An enhanced oncolytic effect was reported with this dual promoter-enabled construct [32].

Other studies have successfully implemented anti-tumor gene therapy using the Ad OV platform to deliver a tumor suppressor gene, TSLC1, which is lost in many human cancers, including liver cancers, and conserved in normal cells [33].

A parallel strategy to enhance tumor-killing effect was achieved by adding lethal mitochondrial genes to the Ad OV platform to be selectively expressed in tumor cells by the vector. One such vector that has been developed encodes the second mitochondria-derived activator of caspases (SMAC) protein. It demonstrated superior oncolytic potency but had also exhibited cytotoxicity toward normal cells, emphasizing the importance of achieving tumor selectivity when deploying more potent vectors [34]. 

Besides AFP, transthyretin [35] has also been identified as an Ad promoter for enhancing HCC vector specificity. Recently, GOLPH2 (GP73), a Golgi protein, was also instituted as an HCC-specific adenovirus promoter [13]. A number of hybrid promoters are also being evaluated. An example of this is the HRE-AFP promoter which has been utilized in an Ad platform to deliver melittin, a bee venom toxic peptide that can induce HCC apoptosis. Inhibitory effects of the vector in HCC were reported as a result of a triple killing mechanism targeting AFP-positive cells in a hypoxic tumor microenvironment, and cells with p53 deficiency [16].

Additional layers of safety through the use of microRNAs, such as let-7 to control vector replication and diminish hepatotoxicity, have also been utilized [36]. Recently, an Ad expressing long non-coding RNA that can competitively bind oncogenic miRNAs has achieved reasonable anti-tumor efficacy [37].

Anti-tumor effect and oncolysis have been improved by a number of strategies. SOCS3, suppressor of cytokine signaling 3, can downregulate Cyclin D1 and anti-apoptotic proteins [38]. Similarly, SOCS1 negatively regulates signal transduction and activation of transcription 3 (STAT3) and can be employed to inhibit STAT3 phosphorylation and ultimately downregulate survivin and c-myc [39]. 

Another strategy to augment OV potency within the tumoral hypoxic microenvironment relies on oxygen-dependent degradation domain-regulated vectors [40]. Similarly, an Ad OV platform using manganese superoxide dismutase has been shown to suppress HCC growth effectively in patient-derived xenografted mice [41].

Recently, the concern regarding the concomitant use of Ad and cisplatin has been overcome by providing XAF1 in a vector platform. XAF1 counters the effects of the inhibitor of apoptosis protein (IAP). This vector enabled enhanced tumor cell apoptosis through activation of the caspase-9/PARP pathway, which ultimately resulted in reduced cisplatin doses [42].

### 3.4. Pancreatic Cancer

Pancreatic cancer has an incidence of 55,440 cases per year and more than 21,000 deaths each year in the USA [20]. Despite recent advances in systemic therapy with the advent of gemcitabine/nab-paclitaxel and FOLFIRINOX, survival rates have not meaningfully changed, and new approaches for treatment are in imminent need.

Efforts are underway to develop specific OVs for pancreatic cancer (Table 6). An Ad with E1B-55 kDa deletion was one of the first adenoviral vectors to show a response to OV comparable to the other tumors. This vector can selectively replicate in *TP53* deficient cells. Additionally, this Ad vector was manipulated to express uracil phosphoribosyl transferase (UPRT), which can enhance therapeutic effects, given that it overcomes 5-fluorouracil resistance [43].

Another gene-therapy-based OV utilized Ad5 carrying suicide genes such as the cytosine deaminase (CD) as well as herpes virus thymidine kinase (HSV TK). This vector was able to improve radiotherapy effect without excessive pancreatic toxicity [44]. Conversely, an Ad carrying the TK gene alone resulted in enhanced survival when used in combination with ganciclovir using an intra-ductal delivery approach in animal models [45].

One approach toward achieving tumor selectivity in pancreatic cancer involves an Ad targeting matrix metalloproteases (MMPs) at the surface entry level and has been shown to reduce metastases with no significant toxicities in vivo for pancreatic cancer models [46].

Likewise, another approach along these lines has employed the use of a *SYE* ligand, with targeting sequence SYENFSA, acting as a pancreatic cancer-targeting ligand capable of boosting promoter specificity of an Ad. Studies conducted with this ligand have achieved robust transduction efficiency, resulting in potent oncolysis in pancreatic tumors [47].

### 3.5. Colorectal Cancer

Colon cancer has an incidence of 97,220 cases per year in the USA, and rectal cancer has an incidence of 43,030 [20]. Despite prevention efforts leading to some improvement in survival due to an increase in early detection, it remains a significant cause of cancer morbidity and mortality. OV therapy represents an exciting avenue for novel therapies in colorectal cancers.

Besides achieving tumor specificity, OV platforms for colon cancer have also focused on expressing pro-drug activating enzymes in cancer cells (Table 7). One early study used the enzyme nitroreductase (NTR) in an E1B-55-kDa deleted Ad. This combination elicited enhanced sensitization of colon cancer cells in vitro to the prodrug CB1954, resulting in reduced tumor growth over five weeks in animal models [48]. Another pro-drug activating enzyme that has been used is carboxypeptidase G2 (CPG2), in an Ad OV platform under the control of an hTERT promoter. Following vector delivery, the administered prodrug ZD2767 was converted by CPG2 into a cytotoxic drug and resulted in tumor growth regression or complete tumor eradication in xenografts [49].

Moreover, the previously described Ad Telomelysin (OBP-301) was modified to yield the construct Telomelysin (OBP-405) that contains an RGD motif in the HI loop of the fiber knob. This modification allows for the ability to overcome the limitation of CAR expression in target cells [50].

Congruent with previous OVs using dual gene virotherapy, the study exhibited promising results in colon cancer cells by using manganese superoxide dismutase as a potential tumor suppressor gene along with the tumor necrosis factor-related apoptosis-inducing ligand (TRAIL) gene embedded in an Ad platform with a deleted E1B-55 kD region. The complete elimination of tumors in xenograft models in vivo was reported in [51].

Furthermore, ST13, a colorectal cancer-specific tumor suppressor gene, was inserted into a CRAds under the control of CEA promoter resulting in significant levels of apoptosis in colon cancer cells [18]. Another study used the colon cancer-specific gene TAp63 in an Ad under the survivin promoter. This construct provided selective replication in HCT116 cells with minimal toxicity in L02 cells [52].

Strategies targeting cancer stem cell/stem cell-like markers are an additional promising approach in OV research. One such construct involved the use of an Ad with a CD133-targeting motif resulting in promising efficacy both in vitro and in vivo [53].

## 4. Clinical Trials and Translational Period of Research

A clinical trial testing Telomelysin in esophageal cancer is eagerly awaited, as the first Ad trial for this type of cancer. As for gastric cancer, some trials have evaluated viral vectors in advanced peritoneal disease, along with ovarian cancer, but none has focused solely on gastric cancer and Ad OV. In the case of pancreatic cancer, primary liver cancers, and colorectal cancer, a number of clinical trials are ongoing (Table 8). Data emerging from these studies will inform the design of the next generation of Ad vectors. It is widely recognized that vector delivery is a challenging step in the eventual application of OV, and advances in this realm are critically needed to address adenoviral liver immune clearance, improve the intra-tumoral viral spread and bystander killing effect, enhance tumor-cell infection, and provide tumor-specific immunity [54].

## 5. Conclusions

Adenoviral vectors represent a promising platform for cancer therapy in GI cancers as highlighted in this review. A number of exciting strategies applied to OV vector design have facilitated tumor selectivity, potent cytotoxicity, and tumor microenvironment modulation. A broad array of pre-clinical evaluations in GI cancers is showing promise, and a number of these concepts have been carried forward to early phase clinical studies. It is anticipated that future efforts will encompass the study of OV Ads in combination with immunotherapies such as immune checkpoint inhibitors, improvements in the vector delivery/evasion of immune response, and amalgamation with gene editing approaches.

## Figures and Tables

**Table 1:** Natural tropism of adenoviral platforms.

Subgroup	Serotype	Attachment Receptors	Natural Tropism
A	12	CAR	Gastrointestinal
B1	35	CD46	Respiratory
B2	3	DSG-2	Renal
B3	11	CD46 and DSG-2	Renal
C	2 and 5	CAR	Respiratory
D	19	CAR and sialic acid	Ocular
E	4	CAR	Respiratory and Ocular
F	40 and 41	CAR	Gastrointestinal
G	52	unknown	Gastrointestinal

CAR: coxsackie-adenovirus receptor; DSG-2: desmoglein-2.

**Table 2:** Adenoviral platform and incorporated promoters for gastrointestinal malignancies.

Virus Promoter	GI Cancer	Ref.
Cytomegalovirus (CMV)	Ubiquitous	[10]
Midkine (MK)	Gastric	[11]
Cyclooxygenase-2 (Cox-2M) and (Cox-2L)	Gastric	[11]
Alpha-fetoprotein (AFP)	HCC	[12]
Golgi protein 73	HCC	[13]
Human telomerase reverse transcriptase (hTERT)	Cancer cells in general	[14]
Survivin	Cancer cells in general	[15]
Chimeric: Hypoxia-response element (HRE) and Alpha-fetoprotein (AFP)	HCC	[16]
Carcinoembryonic antigen (CEA)	Gastric cancer cells and Colon cancer cells	[17,18]

HCC: Hepatocellular carcinoma; GI: Gastrointestinal.

**Table 3:** Preclinical research in esophageal cancer.

Viral Construct Name	In Vitro Cell Line	In Vivo Model	Vector Modifications	Conclusion	Ref.
AxE1AdB	EC-GI-10 T.Tn.	CB17 scid mouse CDX Heterotopic subcutanous transplant	*E1A* gene abolish binding to pRB	Enhanced apoptosis, and cytotoxicity against p53-mutant cells	[21]
Ad/TRAIL-E1	Seg-1 Seg-1 with Radioresistance (R) TE-2, and TE-2R	Nude mice CDX Heterotopic subcutanous transplant	hTERT promoter controlling *E1A*	Ad/TRAIL-E1 preferentially targeted radioresistant-cells	[22]
Ad-delE1B55	TE-1 TE-2 TE-10 TE-11 YES-2 YES-4 YES-5 YES-6 T. Tn	Nude mice CDX Heterotopic subcutanous transplant	CMV Promoter Deleted a part of E3 region and 55 kDa-encoding E1B region	The combinatory antitumor effect depends on the chemotherapy agent	[23]
Telomelysin (OBP-301)	A549 H1299	Nude mice PDX Orthotopic transplant	hTERT Promoter Deleted a part of E3 region and 55 kDa-encoding E1B region	A substantial anti-tumor effect was achieved when radiation followed the intratumoral injection	[24]
Ad-hTERTp-E1a-HN	EC109	Nude mice CDX Heterotopic subcutaneous transplant	hTERT Promoter Deleted a part of E3 region and 55 kDa-encoding E1B region Expressing HN from NDV	Suppression in tumor volume in both delivery modes IT and IVComplete response to vector IT injection	[26]

Abbreviations: Scid: severe combined immunodeficient; CDX: cancer cell-line-derived xenotransplant; PDX: patient-derived xenotransplant; pRB: Retinoblastoma protein; CMV: cytomegalovirus; hTERT: human telomerase reverse transcriptase; TRAIL: TNF-related apoptosis-inducing ligand; HN: hemagglutinin-neuraminidase; NDV: Newcastle disease virus; IT: intratumor; IV: intravenously.

**Table 4:** Preclinical research in gastric cancer.

Viral Construct Name	In Vitro Cell Line	In Vivo Model	Vector Modifications	Conclusion	Ref.
AdCEAp/Rep	MKN-45 MKN-74	-	CEA promoter	Cytotoxicity against CEA producing cells was dose-dependent	[17]
Telomelysin (OBP-301)	MKN-45 MKN-74	NOD/SCID Mice CDX Heterotopic transplant	hTERT promoter	Cell death in quiescent CD133+ cells	[14]
E1B 55-kDa-attenuated Ad	AGS MGc80-3	C57Bl6/J Mice CDX Heterotopic transplant	E1B 55-kDa-deficient Ad expressing Endostatin	Synergistic effect	[27]
AdSurp-Hsp70	SGC-7901 BCG-823 MNK-45	Nude mice CDX Heterotopic transplant	Survivin promoter Vector expressing chaperone Hsp-70	Selective replication in survivin-positive tumor cells	[15]

Abbreviations: CEA: carcinoembryonic antigen; hTERT: human telomerase reverse Transcriptase; NOD/SCID: non-obese diabetic/severe combined immunodeficient; CDX: cancer cell-line-derived xenotransplant.

**Table 5:** Preclinical research in liver cancer.

Viral Construct Name	In Vitro Cell Line	In Vivo Model	Vector Modifications	Conclusion	Ref.
CV890	HepG2 Huh7 Hep3B PLC/PRF/5 SNU449	Nude mice CDX Heterotopic subcutaneous transplant	AFP TRE to control an artificial E1A-IRES-E1B bicistronic cassette in an adenovirus 5 vector	Volume of distant xenografts dropped below baseline at 4 weeks	[29]
CNH500-p53	Hep3B HepG2 SMMC-7721	-	hTERT promoter and HRE promoter	Higher oncolytic effect	[32]
ZD55-Smac	Bel-7404 SMMC7721 Huh-7	-	Incorporation of therapeutic gene: ZD-Smac Under CMV promoter	ZD55-Smac was superior to ONYX-015	[34]
AFP-D55-SOC3	Hep3B PLC HepG2 Huh-7 LM6 BEL7404	Nude mice CDX Heterotopic subcutaneous transplant	SOCS3 downregulate Cyclin D1 and anti-apoptotic proteins such as XIAP, Survivin, Bcl-xL, and Mcl-1	Restoration of SOCS3 antagonize HCC therapeutic resistance to TRAIL	[38]
SD55-TSLC1	Huh-7	Nude mice CDX Heterotopic subcutaneous transplant	Expression of TSLC1 a tumor suppressor gene	Caspase pathways provide antitumor effect	[33]
AdCN305-SOCS1	Bel-7404 Hep3B Huh-7 SMMC7721	Nude mice CDX Heterotopic subcutaneous transplant	Expression of an SOCS1 a negative regulator of STAT3	Inhibition of STAT3 phosphorylation and downregulation of survivin, cyclin-D1, Bcl-xL, and C-myc	[39]
AdCN205-IL-24-miR-34a	PLC/PRF/5 Huh7 Bel-7404	Nude mice CDX Heterotopic subcutaneous transplant	Co-expression of miRNA-34a and IL-24	Complete tumor regression	[36]
QG511-HA-Melittin	Hep3B SMMC7721 HepG2	Nude mice CDX Heterotopic subcutaneous transplant	hybrid promoter, hypoxia-response element and alpha-fetoprotein (HRE)-AFP	Inhibit the growth of HCC xenografts	[16]
AdSVPE1a-lncR	Huh-7 HepG2 SMMC7721 Hep3B L02	Nude mice CDX Heterotopic subcutaneous transplant	Long noncoding RNA expression under a surviving promoter	Competitively consumes OncomiRs (oncogenic miRNAs) promoting tumor shrinkage	[37]

Abbreviations: CDX: cancer cell-line-derived xenotransplant; hTERT: human telomerase reverse transcriptase; HCC: hepatocellular carcinoma; SOCS1: suppressor of cytokine signaling 1; SOCS3: suppressor of cytokine signaling 3; TRAIL: TNF-related apoptosis-inducing ligand.

**Table 6:** Preclinical research in pancreatic cancer.

Viral Construct Name	In Vitro Cell Line	In Vivo Model	Vector Modifications	Conclusion	Ref.
Ad5-yCD/mutTKSR39rep-ADP	Panc 1 MiaPaCa-2	Nude mice CDX Heterotopic subcutaneous transplant; GEMM, CDX Orthotopic transplant	Contains a bacterial cytosine deaminase (CD) and wild-type herpes simplex virus thymidine kinase (HSV-1 TK) gene under CMV promoter	Improved the effectiveness of radiotherapy without excessive toxicity	[44,45]
AdTATMMP	RWP1 Panc1 PSC-21 CAF-25 CAF-28	GEMM CDX orthotopic transplant	AdTATMMP transduction is activated by matrix metalloproteases MMP2 and MMP9	In comparison to Ad5 wild type, there was increased antitumor activity	[46]
AdSur-SYE	AsPC-1 BxPC-3 Panc-1 MIAPaCa-2	Nude mice CDX Heterotopic subcutaneous transplant	Displays a pancreatic cancer targeting sequence SYENFSA on the fiber knob; survivin promoter	High infectivity in human pancreatic cancer tissues	[47]

Abbreviations: GEMM: Genetically engineered mouse model; CDX: cancer cell-line-derived xenotransplant.

**Table 7:** Preclinical research in colorectal cancer.

Viral Construct Name	In Vitro Cell Line	In Vivo Model	Vector Modifications	Conclusion	Ref
CRAd-NTR(PS1217H6)	SW480	Nude mice CDX Heterotopic subcutaneous transplant	Vector E1B-55-KDa-deleted expressing prodrug-activating enzyme nitroreductase (NTR)	Greater sensitization to the prodrug CB1954	[48]
Telomelysin OBP-405	SW620	Nude miceCDX Heterotopic subcutaneous transplant	Vector has mutant fiber containing the RGD peptide, CDCRGDCFC, in the HI loop of the fiber knob	Increased infection property	[50]
ZD55-MnSOD ZD55-TRAIL	SW620	Nude mice CDX Heterotopic Random tumor inoculation	Vector with the E1B 55-kDa gene deletion and expressing Manganese superoxide dismutase (MnSOD)	Effective oncolysis	[51]
AdV.hTERT-CPG2	SW620 SW480 HCT116 LS174T LoVo DLD-1 HT-29 Caco-2 Colo205	Nude mice CDX Heterotopic subcutaneous transplant	Delivery of gene for the prodrug-activating enzyme carboxypeptidase G2 (CPG2) to tumors	significant bystander effects in vivo	[49]
Ad·(ST13)·CEA·E1A(Δ24)	SW620 HT29 HCT116	Nude mice CDX Heterotopic subcutaneous transplant	Vector with CEA promoter expressing suppression of ST13	Induced tumor apoptosis through the mitochondrial-mediated apoptosis pathway	[18]
Ad-survivin-ZD55-TAp63	HCT116	Nude mice CDX Heterotopic subcutaneous transplant	Tap63 expressing cassette in Adenovirus under surviving	in vitro inhibition of cell proliferation	[52]
AdML-TYML	LoVo LS174T	Nude mice CDX Heterotopic subcutaneous transplant	A TYMLSRN peptide motif in place of the primary CAR-binding domains in AB-loop of fiber knob	Selective virus for CSC	[53]

Abbreviations: CDX: cancer cell-line-derived xenotransplant; CEA: carcinoembryonic antigen; CSC: cancer stem-like cells; ST13: tumorigenicity 13.

**Table 8:** Clinical trials using adenoviral oncolytic therapy in gastrointestinal cancers.

GI Cancer	Vector Construct	Phase	Country	Clinicaltrials.gov Number
Pancreatic cancer	Ad5-yCD/mutTKSR39rep-hIL12 (Oncolytic adenovirus expressing two suicide genes and human IL-12)	I	USA	NCT03281382
Pancreatic cancer	LOAd703 Oncolytic adenovirus serotype 5/35 encoding TMZ-CD40L and 4-1BBL	I/II	Sweden	NCT03225989
Pancreatic cancer	LOAd703	I/II	USA	NCT02705196
Pancreatic cancer	VCN-1 expressing PH20 hyaluronidase	I	Spain	NCT02045602
Pancreatic cancer	VCN-1 expressing PH20 hyaluronidase	I	Spain	NCT02045589
Hepatocellular carcinoma	Telomesyn OBP-301	I/II	Korea & Taiwan	NCT02293850
Hepatocellular carcinoma	Recombinant Ad5	III	China	NCT01869088
Liver Cancer	Ad5-CMV-p53	I	USA	NCT00003147
Colorectal cancer	LOAd703	I/II	Sweden	NCT03225989
Colorectal cancer	Ad11/Ad3 Enadenotucirev (previously ColoAd1)	I/II	Belgium & Spain	NCT02028442
